# Treatment of Fracture of the Clavicle

**Published:** 1853-07

**Authors:** W. C. Butler

**Affiliations:** East Avon


### ART. IV.—*Treatment of Fracture of the Clavicle.*

By W. C. Butler, M. D.

That eminent pathologist and distinguished surgeon, Prof. S. D. Gross, in his report on surgery before the State Medical Society of Kentucky for 1852, says: "That a simple, easy, and efficient method of treating fracture of the clavicle, has long been a desideratum with the profession. A vast amount of apparatus has been devised for securing this object, but very little, if any, possesses the requisite fitness or adaptation, while much the greater proportion is absolutely useless—or what is worse, decidedly prejudicial. Notwithstanding his best directed efforts the surgeon but too often finds in these lesions that it is impossible to effect a cure without deformity; much to his own mortification and detriment to his patient, who often remains a cripple for life. Under these circumstances the practitioner should hail with pleasure every suggestion calculated to aid him in the successful management of this class of affections." The talented and able Professor of Surgery in the Buffalo Medical College, holds nearly the same opinion, and gives the preference to the figure of 8 bandage. In speaking of the complicated dressing that had been devised for this lesion, he facetiously remarks to the class, that that of Desault ever put him in mind of the boy who lifted himself over the fence by the strap of his boots.

That truly great surgeon, Prof. Willard Parker, coincides with him in treatment. In speaking of the much that had been written with regard to the application of bandages and dressings, he laid down the following rule for his class: "Gentlemen, see what you want to accomplish and do it. Let the first principles of common sense guide you, and my word for it you will have no cause to regret." He then remarked that any one who knew the anatomical relations of the organs and the relative position of the fractured bone, and could not bring them in juxtaposition and apply a bandage so as to retain them there, was not fit for a surgeon. Such being the views of those whose judgment would almost seem to be law in the matter, it behooves us outside barbarians to inquire, for one moment, why this is so? This is a question of vast importance to the country practitioner, (especially in these days of monomania for instituting suits for mal-practice,) for there is no fracture, so far as my experience goes, half so common.

The clavicle, though far from being straight, may, in one sense, represent a straight line so far as the relative position of organs are concerned. Or in other words, a straight line drawn from the sternal to the humeral end of the clavicle, will represent the relative position the bone must sustain to itself to leave no deformity. This line, from fracture, can only be influenced in two ways: First, by the bones passing each other, thereby making the distance between the extremities shorter than before. Second, in the union that takes place a deviation from said direct line. As in either the transverse or oblique fracture of this bone the same causes are in operation to produce the deformity; viz., the weight of the arm and muscles of the shoulder pulling the humeral end of the clavicle downward and the contraction of the pectoral muscles forward. Also the contraction of the clavicular portion of the sterno cleido mastoidus muscle, drawing the sternal end of the clavicle upward. This is the point, in my humble opinion, which is so much overlooked by surgeons. Suppose for a moment that the sternal end of the fracture, from losing its antagonistic power, is only elevated one line, see how much higher the distal end has to be raised in order to be brought into its proper line. Again; suppose you depress this at this point, three or six lines, and retain it there, see how much less you have to do with "*carrying the shoulder upward and outward*—the latter being sufficient.

If the above views are correct, it demonstrates, conclusively, that we have but two indications: First, to have the humeral end of the clavicle brought sufficiently high to bring it into its proper line with the sternal extremity. Second, to have the length the same as the opposite one. Or, in other words, if we can prevent a union of the bone at any angle, we must of necessity have the shoulder corresponding in height with the other. Third, if we can prevent the fractured ends from passing each other we shall get the length the same as before.

Now let us apply the rule of my valued friend Prof. Parker, and see if this object can be accomplished. Suppose we first put a compress of soft linen six or eight thicknesses, about two inches in length, and wider than the clavicle, directly over the seat of the fracture and let an assistant retain it there. Prepared with strips of adhesive plaster from half an inch to an inch in width and sufficiently long to reach as low or lower than the diaphragm, antero posteriorly, we will let the patient incline to the affected side so as to relax the skin as much as possible; then two assistants with the forearm bare can draw the skin directly up while it is retained with the adhesive straps passing over the compress. These, when thoroughly applied, will fulfill the first indication by bringing the center of the bone down. Let any one incredulous of the power the skin exerts of contracting, under these circumstances, if he has not a case of fracture, have it applied to himself.

The second indication is to be fulfilled with the figure of 8 bandage; and here let me say that I much prefer the simple shoulder braces, properly protected, made of webbing, to the roller. As the shoulder is drawn outward, by the figure of 8 bandage, the pectoral muscle raises under the skin, making your straps more tense and consequently drawing the compress down. This should be borne in mind, and if the length is not satisfactory, carry the elbow across the sternum sufficiently far to obtain the requisite length, and retain it there by a roller passing over the opposite shoulder. And here, again, I prefer a simple pocket handkerchief to the roller.

What says Prof. Gross to this? Is the desideratum of the professor obtained? It certainly fulfills two of his indications, viz., simple and easy, and in my limited practice for the last six years, has the third. Should it possess no other merit, I hope it will be the means of drawing the attention of surgeons into the right field of deduction. My plan has been, when called to see a case of the above fracture, to bring the bones as near juxtaposition as possible, without causing too much inconvenience to the patient, and keep them as near it in the simplest way possible. Then let him pass on from six to ten days, according to the age of the patient, and then put him in the above or permanent dressing.

It is well known to every surgeon that the great difficulty of getting a correct union in this fracture above all others, exists not in bringing the parts in coaptation, but from the restlessness of his patients, especially if a child, of retaining his dressings in situ. So much are some of them impressed with this as the true cause of failure, that a distinguished friend of mine recently remarked to me that he would as soon risk his reputation without a dressing as with one. It is an old saying, that it is a mighty smart cat that can jump out of its own skin. So it must be with a patient that can get away from this dressing.

Think not that I am going to apply to Congress for letters patent, or that I wish you to give this to the profession with the immortal *I* attached to it. Far from it. If every son of Esculapius has not discovered the philosophy of the above treatment, it is no sign he should not, for he has had, or ought to, the same rule to guide him. If any credit is due, give it to my friend, Prof. Parker, who gave the rule which I trust has solved many problems.

East Avon, June 1, 1853.

